# A prognostic nomogram for the cancer-specific survival of white patients with invasive melanoma at BANS sites based on the Surveillance, Epidemiology, and End Results database

**DOI:** 10.3389/fmed.2023.1167742

**Published:** 2023-07-11

**Authors:** Jia-nan Huang, Hai Yu, Yang Wan, Wai-Kit Ming, Fangmin Situ, Leqing Zhu, Yuzhen Jiang, U. Tim Wu, WenHsien Ethan Huang, Wenhui Chen, Jun Lyu, Liehua Deng

**Affiliations:** ^1^Department of Dermatology, The First Affiliated Hospital of Jinan University and Jinan University Institute of Dermatology, Guangzhou, China; ^2^Guangzhou Jnumeso Bio-technology Co., Ltd., Guangzhou, China; ^3^Department of Infectious Diseases and Public Health, Jockey Club College of Veterinary Medicine and Life Sciences, City University of Hong Kong, Hong Kong, Hong Kong SAR, China; ^4^Guangzhou Laboratory, Bioland, Guangzhou, China; ^5^Royal Free Hospital and University College London, London, United Kingdom; ^6^Meng Yi Centre Limited, Macau, Macau SAR, China; ^7^GeneHope Clinic, Taipei, China; ^8^Shanghai Aige Medical Beauty Clinic Co., Ltd. (Agge), Shanghai, China; ^9^Department of Clinical Research, The First Affiliated Hospital of Jinan University, Guangzhou, China; ^10^Guangdong Provincial Key Laboratory of Traditional Chinese Medicine Informatization, Guangzhou, China; ^11^Department of Dermatology, The Fifth Affiliated Hospital of Jinan University, Heyuan, China

**Keywords:** Surveillance, Epidemiology, and End Results database, nomogram, cancer-specific survival, invasive melanoma, BANS sites

## Abstract

**Objective:**

The purpose of this study was to develop a comprehensive nomogram for the cancer-specific survival (CSS) of white patients with invasive melanoma at back, posterior arm, posterior neck, and posterior scalp (BANS) sites and to determine the validity of the nomogram by comparing it with the conventional American Joint Committee on Cancer (AJCC) staging system.

**Methods:**

This study analyzed the patients with invasive melanoma in the Surveillance, Epidemiology, and End Results (SEER) database. R software was used to randomly divide the patients into training and validation cohorts at a ratio of 7:3. Multivariable Cox regression was used to identify predictive variables. The new survival nomogram was compared with the AJCC prognosis model using the concordance index (C-index), area under the receiver operating characteristic (ROC) curve (AUC), net reclassification index (NRI), integrated discrimination index (IDI), calibration plotting, and decision-curve analysis (DCA).

**Results:**

A novel nomogram was established to determine the 3-, 5-, and 8-year CSS probabilities of patients with invasive melanoma. According to the nomogram, the Age at Diagnosis had the greatest influence on CSS in invasive melanoma, followed by Bone Metastasis, AJCC, Stage, Liver Metastasis, Histologic Subtype, Brain Metastasis, Ulceration, and Primary Site. The nomogram had a higher C-index than the AJCC staging system in both the training (0.850 versus 0.799) and validation (0.829 versus 0.783) cohorts. Calibration plotting demonstrated that the model had good calibration ability. The nomogram outperformed the AJCC staging system in terms of AUC, NRI, IDI, and DCA.

**Conclusion:**

This was the first study to develop and evaluate a comprehensive nomogram for the CSS of white patients with invasive melanoma at BANS sites using the SEER database. The novel nomogram can assist clinical staff in predicting the 3-, 5-, and 8-year CSS probabilities of patients with invasive melanoma more accurately than can the AJCC staging system.

## 1. Introduction

Malignant melanoma (MM) is the skin cancer with the highest absolute number of deaths. MM mortality has increased overall over the last 30 years (1). In the United States, the incidence of invasive melanoma has increased over the last 40 years (2), and skin melanoma was the third most prevalent cancer among males in 2019 (3). Further, studies have projected that melanoma incidence rates will continue to rise in the United States (2). A fact sheet from the National Cancer Institute based on the Surveillance, Epidemiology, and End Results (SEER) database indicated that there were an estimated 99,780 new melanoma cases in 2022, which accounted for 5.2% of all new cancer cases, and a death toll of up to 7,650. Meanwhile, the SEER database^1^ indicates that invasive melanoma has higher morbidity and mortality rates among white patients than among those of other races.

Invasive melanoma at back, posterior arm, posterior neck, and posterior scalp (BANS) sites has a worse prognosis than that at non-BANS sites due to sun exposure (4), and UV light exposure is the most significant environmental risk factor (5). Early recognition and follow-up of white patients with invasive melanoma at BANS sites is therefore critical.

Previous studies demonstrated that identifying the primary histologic subtype played a critical role in the prognoses of patients with invasive melanoma (6). The World Health Organization classifies invasive melanoma into four subtypes: nodular melanoma (NM), lentigo maligna melanoma (LMM), superficial spreading melanoma (SSM), and acral melanoma (AM) (7, 8). Some prognostic nomograms have been developed to improve the accuracy of predicting survival in patients with NM (9) or AM (10). However, a comprehensive prediction model for the early diagnosis and prognosis of the four major subtypes of invasive melanoma is still yet to be developed.

The American Joint Committee on Cancer (AJCC) staging system was established to provide standard tumor, node, and metastasis (TNM) categories and stage groupings that can be used to make appropriate clinical decisions (11). While TNM staging has evolved to more accurately reflect patient prognosis, it cannot always predict patient survival (12). Given the limitations of the AJCC staging system, it is necessary to develop a comprehensive prediction model that is based on more-accurate prognostic information.

The nomogram was based on logistic or Cox regression, which is commonly used to predict cancer prognosis (13, 14). The potential independent factors associated with prognosis were investigated in the SEER database. The purpose of this study was to develop and validate a comprehensive prediction model to predict the 3-, 5- and 8-year cancer-specific survival (CSS) rates of individual patients with invasive melanoma (15).

## 2. Methods

### 2.1. Data source and data selection criteria

The SEER database provided us with detailed data on white patients diagnosed with invasive melanoma during 2010–2015 (16). The clinical data were selected from the latest database, designated as “SEER Research Plus Data, 17 Registries, Nov 2021 Sub (2000–2019),” which included Age at Diagnosis, Sex, Marital Status, Rural–Urban Continuum, Primary Sites, Stage, Histologic Subtype, Breslow Thickness, Ulceration, LDH Pretreatment Level, Mitotic Rate, AJCC, Regional Node Status, Bone Metastasis, Brain Metastasis, Liver Metastasis, Lung Metastasis, Survival time, and Survival status.

It is worth noting that the primary sites of invasive MM were BANS, which are coded in SEER as “C44.4-Skin of scalp and neck, C44.5-Skin of trunk, and C44.6-Skin of upper limb and shoulder.” The seventh edition of the AJCC staging system was applied to the patients in this database, and patients diagnosed according to the sixth edition were also converted to the seventh edition (11). Patients were classified into three subgroups for the stage of lymph node metastasis: localized, regional, and distant. The survival statuses were divided into two groups: “dead attributed to this cancer” and “alive/dead attributed to others.” Patients with a diagnosis at autopsy or death certificate only or those with incomplete data on certain variables (race, age, and cause of death) or those who lost to follow-up were excluded.

Because the SEER*Stat statistical software^2^ provides all of the data we analyzed and is available to the public worldwide, we did not need to obtain patient consent or the approval of an institutional review committee for this study.

### 2.2. Statistical analysis

The patients with invasive cutaneous melanoma were selected according to the inclusion and exclusion criteria of our study. For the construction and validation of the prognostic nomogram, all patients were randomly divided into training and validation cohorts at a ratio of 7:3. The log-rank test was also used to determine that there were no significant differences between the training and validation cohorts (*p* > 0.05).

Multivariable Cox regression was used to identify predictive variables associated with CSS (17). Descriptive statistics were applied to both the training and validation cohorts to illustrate the demographic and clinical characteristics of the patients. Median and interquartile-range values were used for Age at Diagnosis, while percentages were used for other categorical variables. In addition to calculating the hazard ratios (HRs) and 95% confidence intervals (CIs) for the variables, a prognostic nomogram was constructed to predict the 3-, 5-, and 8-year CSS probabilities of patients with invasive cutaneous melanoma (18).

We used a series of indicators to evaluate the predictive accuracy of the nomogram after it was established (19). We first evaluated the discrimination performance using the concordance index (C-index) and the area under the receiver operating characteristic (ROC) curve (AUC) (20, 21). The net reclassification index (NRI) and integrated discrimination index (IDI) were then calculated to determine the extent to which the predictive accuracy of the nomogram was better than that of the traditional AJCC staging system (22). Calibration plotting was also used to assess the agreement between predicted probabilities and observed outcomes (23), which was performed using bootstrapping with 500 resamples. Finally, decision-curve analysis (DCA) was conducted to validate the clinical value and utility of the nomogram (24, 25).

All statistical analyzes were performed using R software (version 4.2.1).^3^ R is a free software environment for statistical computing and graphics. Two-sided analyzes were performed, with *p* < 0.05 considered indicative of a significant difference. The TRIPOD Statement aims to improve the transparency of the reporting of a prediction model study (26). TRIPOD checklist was provided in Supplementary material.

## 3. Results

### 3.1. Characteristics of the included patients

The 2,356 eligible patients were randomly divided into the training (*n* = 1,649) and validation (*n* = 707) cohorts. We then depicted the detail information about the demographic and clinical characteristics of patients in different cohorts. The median age at diagnosis was 62 years in both two cohorts (interquartile range = 51–71 years in the training cohort and 51–73 years in the validation cohort). The proportions of patients at AJCC I, II, III, and IV were 56.7, 21.0, 19.0, and 3.2% in the training cohort, respectively, and 56.6, 21.9, 18.5, and 3.0% in the validation cohort. The most common primary site was the trunk (45.8 and 46.1% in the training and validation cohorts, respectively), followed by upper limb/shoulder (37.2 and 39.5%), and scalp/neck (17.0 and 14.4%). Most patients had no bone metastasis (99.2 and 99.4% in the training and validation cohorts, respectively), brain metastasis (99.3 and 99.0%), liver metastasis (99.1 and 99.3%), or ulceration (73.7 and 75.2%). Regarding lymph node metastasis, patients with localized, regional, and distant stages comprised 75.8, 19.8, and 4.4% of the training cohort, respectively, and 76.1, 19.7, and 4.2% of the validation cohort. The most common histologic type was SSM (63.6 and 63.8% in the training and validation cohorts, respectively), followed by NM (28.1 and 29.0%), LMM (7.1 and 6.6%), and AM (1.2 and 0.6%; Table 1).

**Table 1:** The demographic and clinical characteristics of patients in different cohorts.

Variable	Training cohort	Validation cohort
Number of patients *n* (%)	1,649 (70)	707 (30)
Age at diagnosis	62 (51–71)	62 (51–73)
Bone metastasis *n* (%)		
No	1,636 (99.2)	703 (99.4)
Yes	13 (0.8)	4 (0.6)
Brain metastasis *n* (%)		
No	1,638 (99.3)	700 (99.0)
Yes	11 (0.7)	7 (1.0)
Liver metastasis *n* (%)		
No	1,634 (99.1)	702 (99.3)
Yes	15 (0.9)	5 (0.7)
Stage *n* (%)		
Localized	1,250 (75.8)	538 (76.1)
Regional	327 (19.8)	139 (19.7)
Distant	72 (4.4)	30 (4.2)
AJCC *n* (%)		
I	935 (56.7)	400 (56.6)
II	347 (21.0)	155 (21.9)
III	314 (19.0)	131 (18.5)
IV	53 (3.2)	21 (3.0)
Primary Site *n* (%)		
Skin of upper limb and shoulder	613 (37.2)	279 (39.5)
Skin of trunk	755 (45.8)	326 (46.1)
Skin of scalp and neck	281 (17.0)	102 (14.4)
Histologic subtype *n* (%)		
LMM	117 (7.1)	47 (6.6)
SSM	1,049 (63.6)	451 (63.8)
NM	464 (28.1)	205 (29.0)
AM	19 (1.2)	4 (0.6)
Ulceration *n* (%)		
No	1,215 (73.7)	532 (75.2)
Yes	434 (26.3)	175 (24.8)

NM, Nodular melanoma; LMM, Lentigo maligna melanoma; SSM, Superficial spreading melanoma; AM, Acral melanoma.

### 3.2. Variable screening and nomogram establishment

Age at Diagnosis, Primary Site, AJCC, Brain Metastasis, Liver Metastasis, Bone Metastasis, Ulceration, Stage, and Histological Subtype were selected for inclusion in the multivariable Cox regression analysis (17). The analysis indicated that the following variables were significant: Age at Diagnosis (HR = 1.033, *p* < 0.001), Bone Metastasis (HR = 2.758, *p* < 0.01 versus no Bone Metastasis), Brain Metastasis (HR = 2.517, *p* < 0.05 versus no Brain Metastasis), Liver Metastasis (HR = 4.203, *p* < 0.001 versus no Liver Metastasis), and Ulceration (HR = 1.525, *p* < 0.01 versus no Ulceration). In terms of the Stage and AJCC, Regional (HR = 2.202, *p* < 0.01 versus Localized), Distant (HR = 3.904, *p* < 0.01 versus Localized), AJCC II (HR = 1.753, *p* < 0.05 versus AJCC I), AJCC III (HR = 2.941, *p* < 0.01 versus AJCC I), and AJCC IV (HR = 3.197, *p* < 0.05 versus AJCC I). The significant primary melanoma sites were the trunk (HR = 1.563, *p* < 0.01 versus upper limbs and shoulder), and scalp/neck (HR = 1.767, p < 0.01 versus upper limbs and shoulder). The significant histologic subtypes were SSM (HR = 0.966, *p* = 0.919 versus LMM), NM (HR = 1.491, *p* = 0.245 versus LMM), and AM (HR = 2.671, *p* < 0.05 versus LMM; Table 2).

**Table 2:** Selected variables by multivariable Cox regression analysis.

Variable	Multivariable analysis		
	HR	95% CI	*P* value
Age at diagnosis	1.033	1.023–1.043	<0.001
Bone metastasis			
No	Reference		
Yes	2.758	1.300–5.852	<0.01
Brain metastasis			
No	Reference		
Yes	2.517	1.108–5.72	<0.05
Liver metastasis			
No	Reference		
Yes	4.203	2.035–8.679	<0.001
Stage			
Localized	Reference		
Regional	2.202	1.263–3.837	<0.01
Distant	3.904	1.697–8.981	<0.01
AJCC			
I	Reference		
II	1.753	1.086–2.831	<0.05
III	2.941	1.492–5.798	<0.01
IV	3.197	1.149–8.895	<0.05
Primary site			
Skin of upper limb and shoulder	Reference		
Skin of trunk	1.563	1.132–2.158	<0.01
Skin of scalp and neck	1.767	1.223–2.555	<0.01
Histologic subtype			
LMM	Reference		
SSM	0.966	0.500–1.869	0.919
NM	1.491	0.760–2.925	0.245
AM	2.671	1.005–7.097	<0.05
Ulceration			
No	Reference		
Yes	1.525	1.153–2.016	<0.01

NM, Nodular melanoma; LMM, Lentigo maligna melanoma; SSM, Superficial spreading melanoma; AM, Acral melanoma.

**Figure 1:**
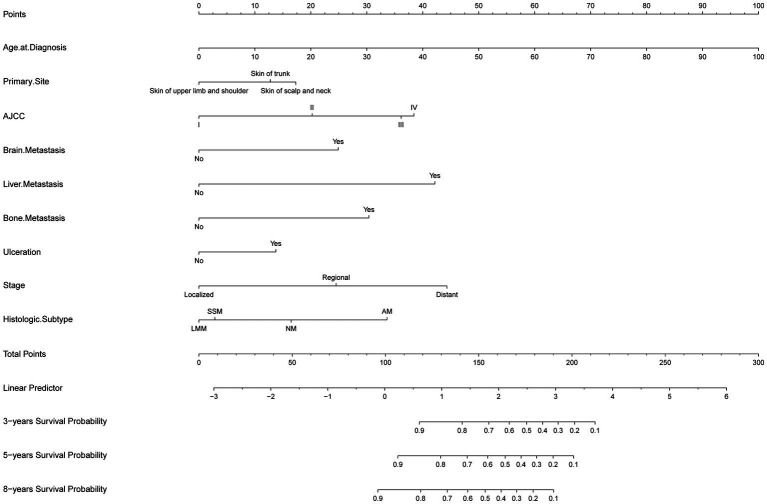
The developed nomogram indicates that the Age at Diagnosis has the greatest influence on CSS in invasive MM, followed by the Bone Metastasis, AJCC, Stage, Liver Metastasis, Histologic Subtype, Brain Metastasis, Ulceration, and Primary Site.

A nomogram for predicting 3-, 5-, and 8-year CSS probabilities was developed based on the identified significant variables (14). To use the nomogram, a score is first assigned to each variable on a point scale. The total score is then calculated by adding the scores for all variables, and a vertical line is drawn down from the total-points row to estimate the 3-, 5-, and 8-year survival rates. A worse prognosis was associated with a higher total score. The developed nomogram indicates that the Age at Diagnosis has the greatest influence on CSS in invasive MM, followed by the Bone Metastasis, AJCC, Stage, Liver Metastasis, Histologic Subtype, Brain Metastasis, Ulceration, and Primary Site (Figure 1).

### 3.3. Nomogram comparison and evaluation

**Figure 2:**
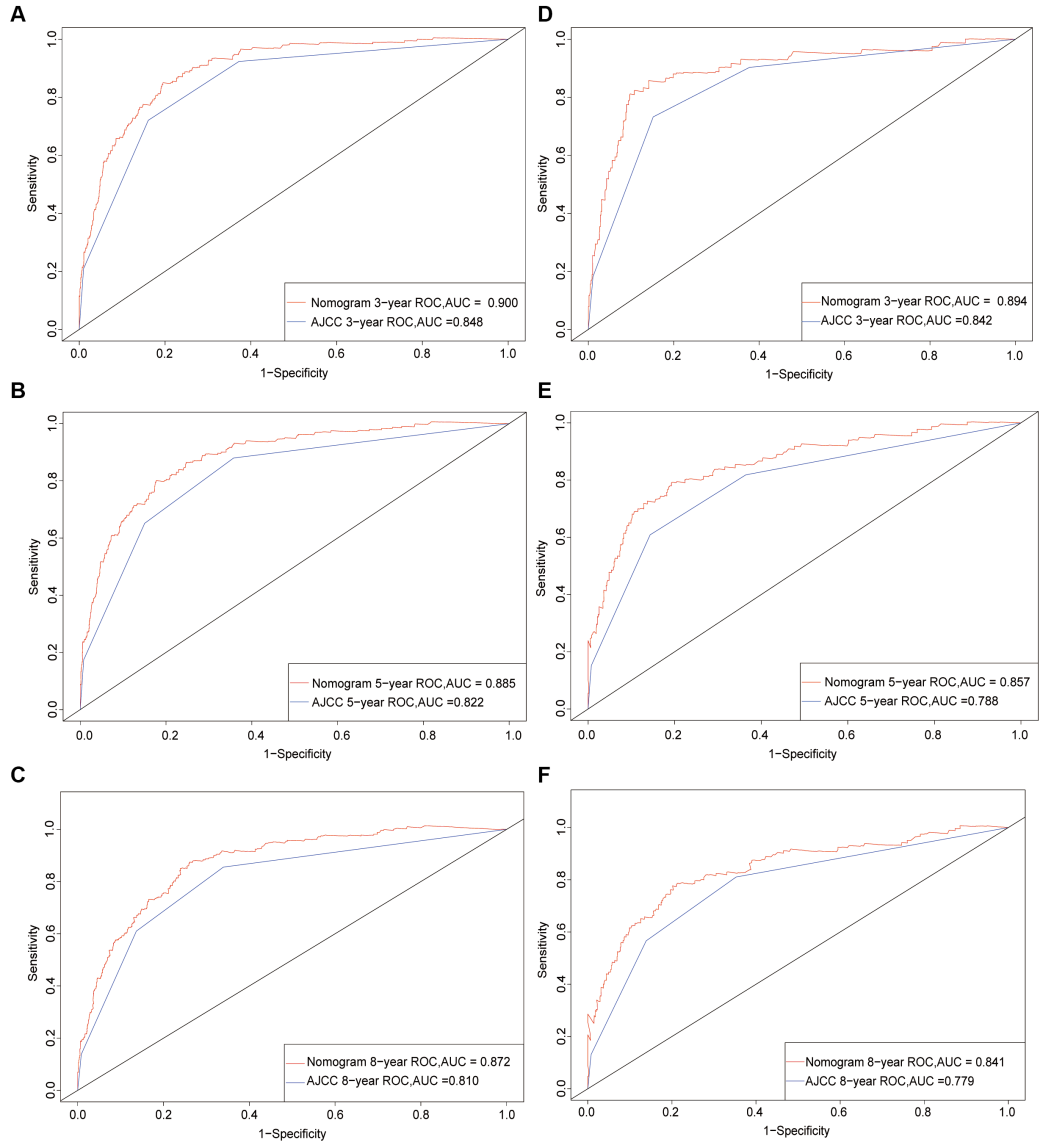
ROC curves. The area under the receiver operating characteristic (ROC) curve (AUC) was used to evaluate the performance of the new nomogram. **(A–C)** ROC curve for the training cohort; **(D–F)** ROC curve for the validation cohort.

Following the establishment of the prognostic nomogram, we used a set of indicators to assess its performance. We first used the C-index to assess the discrimination of the nomogram (21). A C-index of 0.5 indicates that there is no discrimination, whereas 1.0 indicates that patients with different outcomes are perfectly separated; a higher C-index indicates a better predictive ability. We found that the C-index of the nomogram was higher than that of the AJCC staging system in both the training (0.850 versus 0.799) and validation (0.829 versus 0.783) cohorts. Both C-index and AUC ranged from 0.5 to 1. We further compared ROC curves (20), and found that the AUC values of the nomogram at 3, 5, and 8 years (0.900, 0.885, and 0.872, respectively, in the training cohort, and 0.894, 0.857, and 0.841 in the validation cohort) were higher than those of the AJCC staging system (0.848, 0.822, 0.810, 0.842, 0.788, and 0.779, respectively). These results indicated that the nomogram had a better predictive ability than did the seventh edition of the AJCC staging system for 3-, 5-, and 8-year CSS in both the training and validation cohorts (Figure 2).

The NRI and IDI were used to compare the accuracies of the two models to determine the improvement obtained (22). The NRI values for the 3-, 5-, and 8-year CSS probabilities were 0.411 (95% CI = 0.283–0.523), 0.416 (95% CI = 0.292–0.517), and 0.310 (95% CI = 0.180–0.451), respectively, in the training cohort, and 0.411 (95% CI = 0.159–0.594), 0.338 (95% CI = 0.129–0.545), and 0.321 (95% CI = 0.111–0.532) in the validation cohort. In addition, the IDI values for the 3-, 5-, and 8-year CSS probabilities were 0.054, 0.061, and 0.068, respectively (*p* < 0.001), in the training cohort, and 0.053, 0.062, and 0.069 (*p* < 0.001) in the validation cohort. All of the NRI and IDI values being greater than zero indicated that the new model had superior predictive ability.

**Figure 3:**
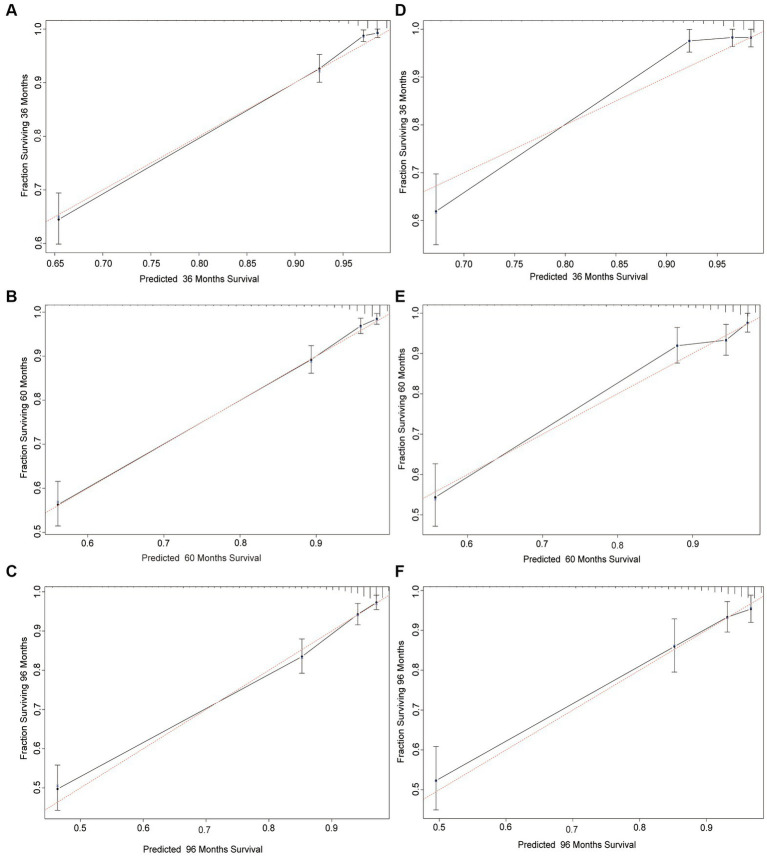
Calibration plots of the nomogram for predicting 3-, 5-, and 8-year CSS probability in invasive melanoma patients. Calibration curves reflect the relationship between the predicted probabilities and actual values of the training cohort **(A–C)** and validation cohort **(D–F)**.

Calibration plots indicated that the standard curves of the 3-, 5-, and 8-year CSS probabilities in the nomogram were very close to the standard 45-degree diagonal lines, indicating that it was well calibrated (23). The calibration plots demonstrated excellent consistency between the predicted probabilities and the observed outcomes in the training and validation cohorts for 3-, 5-, and 8-year CSS. These findings suggested that the nomogram was highly reliable (Figure 3).

**Figure 4:**
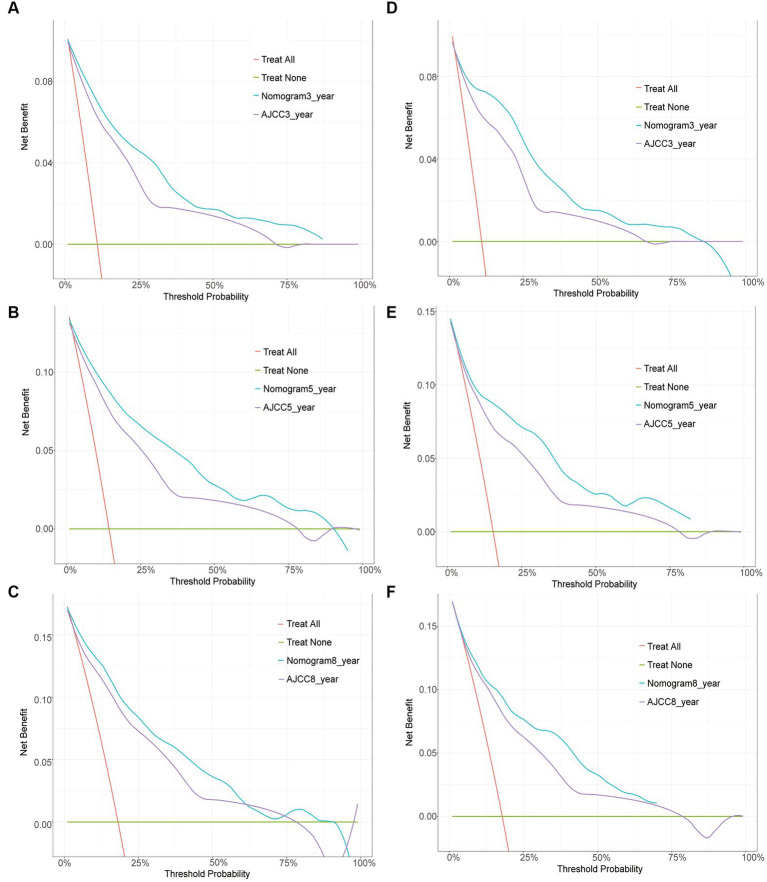
Decision curve analysis. Curves of the 3-, 5-, and 8-year CSS probability in patients with invasive melanoma. The abscissa represents the threshold probability, while the ordinate represents the net benefit rate. The horizontal line denotes that all samples are negative and that none are treated, resulting in a net benefit of zero. All samples are positive, as indicated by the oblique line. A negative slope represents the net benefit. **(A–C)** DCA curve for the training cohort; **(D–F)** DCA curve for the validation cohort.

The clinical utility of the predictive models was evaluated using DCA curves, which calculated the net benefit at various threshold probabilities. In DCA curves, the abscissa represents the threshold probability and the ordinate represents the net benefit after it is subtracted from the disadvantage (24, 25). Although both models yielded net benefits when compared with the AJCC staging system, the 3-, 5-, and 8-year DCA curves of the nomogram were found to be enhanced in both the training and validation cohorts, indicating that the nomogram had favorable clinical utility (Figure 4).

## 4. Discussion

Melanoma morbidity has increased in the United States over the last 50 years owing to an aging population and high age-specific rates in the elderly, according to the findings of a study that used age-period-cohort models to describe current trends and project future incidence rates and occurrences of melanomas until 2031 (27). The AJCC stage is known to be correlated with survival in patients with melanoma (11). The 5-year melanoma-specific survival rate ranges from as high as 99% in patients with stage I disease to less than 10% in some patients with stage IV (distant metastatic) disease (28).

A previous study indicated that invasive melanoma evolves from precursor lesions through sequential genetic alterations and that UV radiation exposure is a major factor in the development and progression of melanoma (29). Invasive melanomas at BANS sites therefore have poor prognoses, particularly for lesions on the scalp/neck. Most studies of cutaneous head and neck melanomas have found that they are associated with a lower survival rate than those at other sites (30). Scalp melanomas have more aggressive clinicopathologic features and mortality rates more than twice that of melanomas located elsewhere (31). In brief, the anatomic location of melanoma has been found to independently influence melanoma-specific survival (4).

Moreover, the primary histologic subtype of cutaneous melanoma has been found to influence prognosis and prediction (6). The principal subtypes are SSM, NM, LMM, and AM. NM is clinically unique and has been found to be the leading cause of melanoma-related death (32). AM is a rare form comprising approximately 3% of all melanomas, which affects the worldwide population regardless of skin color and has a worse survival rate than other cutaneous melanomas (33).

Combined with the results of another study, we discovered that AM was a risk factor that reduced the survival rate of invasive melanoma, and we speculated that this could be because patients with AM are more likely to develop sentinel lymph node metastases from MM (34). Another retrospective, observational cohort study demonstrated that patients with invasive melanoma with regional lymph node or distant metastases tended to have poor survival outcomes (35). Based on a review of nearly 2 decades of data, we found that lymph node metastasis was a major predictor of outcome in patients with melanoma (36).

Patients with ulcers had a lower survival rate in the present study, which was consistent with previous research. Previous findings on outcomes support the current melanoma staging system by demonstrating that ulceration is significantly predictive of poor survival (37).

The results indicated that types of metastases other than lung metastasis were suitable for inclusion in the model. Previous research has indicated that bone, liver, and brain metastases all significantly contribute to disease-related morbidity and mortality (38–41). Bone is the third most common site of metastasis for a wide range of solid tumors, including melanoma, and cancer is rarely cured once it has spread to the bone (38). Liver metastasis continues to be a major impediment to successful cancer management, particularly in certain cancers such as melanoma (39). Melanoma brain metastases are common and have a particularly poor prognosis; they are the direct cause of death in 60–70% of patients with melanoma (40). In summary, patients with liver, brain, or bone metastases as the only disease site had shorter survival than those with metastases at other sites (41).

Our new model included the factors mentioned above, including Age at Diagnosis, AJCC, Primary Site, Histologic Subtype, Stage, Ulceration, Bone Metastasis, Liver Metastasis and Brain Metastasis. We used these factors to construct the new model for several reasons (1): the nomogram provided higher C-index and AUC values than the AJCC staging system in both the training and validation cohorts (2), the calibration plots demonstrated a greater consistency between the actual observations and predicted probabilities of 3-, 5-, and 8-year CSS (3), DCA indicated that the new model yielded net benefits that were greater than those of the traditional AJCC staging system in both the training and validation cohorts, and (4) all of the NRI and IDI values being greater than zero indicated that the new model had superior predictive ability.

However, there were limitations to our study. It had a small sample and a retrospective design, resulting in unavoidable selection bias. Another limitation was that our study variables did not include potential important factors such as the family history and the average duration of sun exposure per day. Furthermore, we focused on specific variables aimed at identifying predictive factors through relevant clinical indicators in a large population and establishing a predictive model for preoperative prediction to reduce unnecessary invasiveness. The predictive model needs to be combined with the clinical physician’s actual situation in decision-making during actual application. Clinical physicians can comprehensively evaluate and make decisions based on the predictive results of the clinical prediction model and the specific situation of the patient, such as radiation and chemotherapy information, treatment timing information, surgical methods, etc. In practice, we should consider other potential factors to improve our predictive model and conduct further analysis in future studies to explore the potential impact of these factors on our research results.

## 5. Conclusion

This study was the first to establish a comprehensive nomogram for the CSS of white patients with invasive melanoma at BANS sites based on the SEER database and to evaluate it using a series of indicators. Our novel nomogram can assist clinical staff in predicting the 3-, 5-, and 8-year CSS probabilities of patients with invasive melanoma at BANS sites more accurately than the AJCC staging system.

## Data availability statement

The original contributions presented in the study are included in the article/Supplementary material, further inquiries can be directed to the corresponding authors.

## Ethics statement

The principles articulated in the 1964 Declaration of Helsinki and its later revisions guided every method carried out for this investigation. Since all patient data in this investigation were de-identified and the SEER research data are publically available, informed permission and institutional review board approval were not necessary.

## Author contributions

All authors listed have made a substantial, direct, and intellectual contribution to the work and approved it for publication.

## Funding

The study was supported by Key Scientific Problems and Medical Technical Problems Research Project of China Medical Education Association (2022KTZ009) and Guangdong Provincial Key Laboratory of Traditional Chinese Medicine Informatization (2021B1212040007).

## Acknowledgments

We thank the Surveillance, Epidemiology, and End Results (SEER) Program registries for their contributions to the SEER database.

## Conflict of interest

YW was employed by Guangzhou Jnumeso Bio-technology Co., Ltd. LZ was employed by Bioland. UW was employed by Meng Yi Centre Limited. WC was employed by Shanghai Aige Medical Beauty Clinic Co., Ltd. (Agge).

The remaining authors declare that the research was conducted in the absence of any commercial or financial relationships that could be construed as a potential conflict of interest.

## Publisher’s note

All claims expressed in this article are solely those of the authors and do not necessarily represent those of their affiliated organizations, or those of the publisher, the editors and the reviewers. Any product that may be evaluated in this article, or claim that may be made by its manufacturer, is not guaranteed or endorsed by the publisher.

## Footnotes

^1^ http://seer.cancer.gov/statfacts/html/melan.html

^2^ http://seer.cancer.gov//seerstat/

^3^ http://www.r-project.org
